# Nanocarbon Type Xerogel Materials Designed for Water Desalination

**DOI:** 10.3390/ma14174932

**Published:** 2021-08-30

**Authors:** Gabriela Hristea, Mihai Iordoc, Andreea Culcea

**Affiliations:** Environment, Energy & Climate Change Department, National Institute for Research and Development in Electrical Engineering ICPE-CA, Splaiul Unirii 313, 030138 Bucharest, Romania; mihai.iordoc@icpe-ca.ro (M.I.); andreea.culcea@icpe-ca.ro (A.C.)

**Keywords:** water desalination, water deionization, carbon xerogels, nanocarbons, capacitive deionzation, resorcinol-formaldehyde gels, carbon whiskers, nanowhiskers, electrodes

## Abstract

The relative performance of different porous solids in different applications is highly dependent on the internal pore structure of each material. Highly porous carbon materials can be prepared by evaporative drying and the pyrolysis of resorcinol-formaldehyde gels. By determining the correct synthesis parameters, the pore system of such materials can be reshaped. Depending on some important processing factors such as the dilution ratio or the initial pH of the precursor solution, various porous or non-porous carbon materials can be synthesized. This paper addresses carbon xerogels (CX) designed as a material electrode in capacitive deionization (CDI) systems for water desalination. In this work CX materials were synthesized via poly-condensation reactions of resorcinol with formaldehyde (RF) on a carbon felt sheet followed by pyrolysis. The resulting sheets were used as electrodes to develop a CDI experimental multi-cell laboratory system. The initial pH of the RF solution and the dilution ratio effect on the resulting carbon surface area and structure were analyzed. Surface area measurements using the BET method and an electrochemical capacitance evaluation of the obtained xerogels through electrochemical impedance spectroscopy were also performed. Finally, using our experimental CDI multi-cell laboratory system based on the obtained CX, we discuss the experimental data for the desalination rate as a function of the voltage and salt concentration. As a result, the developed model’s efficiency is demonstrated. The main goal of this work was to develop an efficient electrode-based novel carbon that could be commercially competitive, as well as to create guidelines for future desalination research using CX electrode materials.

## 1. Introduction

The desalination of seawater based on capacitive deionization (CDI) principles is a unique and novel technology. It seems straightforward that charged ionic species can be easily removed from aqueous solutions with this feasible method.

CDI has developed itself as a viable alternative to reverse osmosis, electrodialysis, and thermal distillation for desalination [1,2,3]. It makes use of the basic capacitor principles to remove dissolved ions from a water stream. The electrodes happen to be the electrochemical surfaces where ions are adsorbed following the principles of the double layer [4]. Therefore, CDI can be considered as an electrochemical reaction that occurs in a “flow-through” double layer capacitor [5]. During CDI treatment, salty water flows between pairs of porous charged electrodes [6]. The strong electrostatic forces between the ionic species in the solution and the charged electrodes control CDI separation. Deionized water is generated when positive and negative electrodes attract oppositely charged ions. After being saturated with salts or impurities, the electrodes are regenerated by removing the electric field when the adsorbed ions on the electrodes’ surfaces are desorbed and released into a concentrated stream. Previous research demonstrated that the efficiency of CDI strongly depends upon the surface property of the electrodes, such as surface area, the size distribution of pores, the microstructure, chemical functional groups, or the electro-sorption capacitance [7,8,9,10]. However, operational parameters such as the external voltage, the mass flow, the interlayer thickness between the two electrodes, the flow channel design, or the concentration of the solution of ions, among others, influence CDI’s desalting performance [11].

The adsorption and desorption of ions is complete during the charging–discharging of the CDI electrodes. Therefore, CDI technology merely requires electrode materials with a high surface area, a high capacitance, and a high desalination efficiency [12]. Recently, carbon materials such as carbon nanotubes [13], graphene [14], aerogels [15,16], xerogels [17], and activated [18] or mesoporous carbons [19], have been used in the fabrication of tailored electrodes engineered for this technology [20]. However, the most frequently used electrode material is carbon aerogel [21,22,23,24]. It is common practice to produce carbon aerogels by the supercritical drying of wet resorcinol–formaldehyde (RF) gels. Even so, this method requires high pressures (up to 6.89 × 10^6^ Pa), which could generate potential hazards [25,26,27] and make it expensive to scale-up production for industrial use.

It is advantageous to make the electrodes ion-selective, to improve the charge efficiency, and thus reduce the energy input corresponding to a proper desalination performance. This can be usually completed by chemically modifying the electrodes [28,29,30,31,32,33,34,35]. Non-modified carbons can adsorb either cations or anions, depending on the electrode potential [16,36,37]. The desalination of water can be achieved using a system design based on these non-modified carbons. CDI has the potential to play a significant role in the water industry, despite the challenges [38,39,40,41]. However, capital, and operational costs are major concerns for CDI given that, its main competitive advantage is the ability to remove a wide spectrum of ionic species with high recovery rates. In part, today’s high costs are due to the low production volumes and immature industrial processability of electrodes. While CDI membrane development has received relatively little attention, significant essential emphasis has been placed on novel carbon materials for use in CDI.

In this research, we generate carbon nanowhiskers as a new form of carbon xerogels (CX) from resorcinol–formaldehyde precursors and successfully use them as electrodes in a CDI experimental laboratory system. The usual methods to grow carbon nanowhiskers include plasma-enhanced hot filament chemical vapour deposition, plasma-enhanced chemical vapour deposition, electron-beam-induced deposition, field emission-induced growth, dry plasma processing, or ion beam delivery. Furthermore, metals are thought to catalyse the growth of whisker structures [42]. In this study, carbon (nano)whiskers were produced by the polycondensation reaction of resorcinol with formaldehyde, without the use of an external carbon source (as in a plasma-controlled environment) or a metal catalyst. In this light, we consider our method of generating carbon (nano)whiskers as a valuable alternative synthesis technique to the ones already or currently in use. Assuming that CX properties can be tailored depending on the synthesis conditions, their modeling for CDI systems was based on their relatively high electrical conductivity, which makes them ideal materials for use as electrodes in energy storage devices, as long as CDI has been assimilated with an electrical double layer capacitor [43]. Laboratory trials confirm that carbon (nano)whiskers can compete from an operational perspective with well-known porous carbon xerogels.

## 2. Materials and Methods

For the synthesis of the RF gels, resorcinol [C_6_H_4_(OH)_2_](R) (Wako Pure Chemical Industries, research grade) and formaldehyde 37% (HCHO) (F), (Wako Pure Chemical Industries, research grade) were purchased from Merck, Darmstadt, Germany, Chimexim, Bucharest, Romania. The catalyst used was sodium carbonate (C) Na_2_CO_3_ (99%, Merck KGaA, Darmstadt, Germany). Anhydrous glycerin (pure grade) Chemical Company, Iasi, Romania, acetone 99.5%, and ethanol 99% from Redox Research & Analytics SRL Otopeni, Romania were also used as reagents. Deionized water was used as a solvent. The processing of the CX electrodes followed the path described below.

### 2.1. Synthesis of the Initial RF Solution

In this work, the RF organic gels were synthesized by the reaction of resorcinol (R) with formaldehyde (F), (2:1 molar ratio) in the presence of sodium carbonate Na_2_CO_3_ as a catalyst and water. To prevent the rapid increase in the polycondensation rate between the reagents, pure glycerin was added as a plasticizer agent (1%wt).

The amount of the catalyst was considered as the molar ratio of resorcinol to the catalyst (R/C), whose value was chosen from R/C = 20 to 1000 [44,45,46,47,48,49,50,51,52]. Next, RF solutions were immersed in acetone media, poured into glass vials, and heated between 50 and 70 °C for up to 180 min in reflux system conditions.

### 2.2. Preparing the CX Sheets Electrodes—Design of the RF Components/Electrodes for the CDI Experimental Module

In terms of our intended application, some disadvantages of carbon gels include brittleness and shrinkage during processing. To address this issue, it has been demonstrated that carbon aerogels can be reinforced with carbon fibers to reduce shrinkage and increase flexibility [53]. By impregnating the RF solution in carbon fiber type materials and then drying/pyrolyzing them, a flexible carbon gel can be prepared. Our approach to obtaining CDI electrodes based on CX followed the same path.

**Figure 1:**
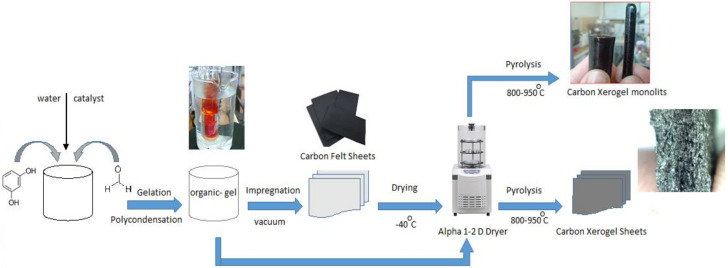
Schematic representation of the overall CX sheets electrodes synthesis process.

In brief, several steps were involved in our method of preparing CX electrodes: a-synthesis of the initial RF organic solutions, b-gelation, c-drying, d-impregnation of carbon felt sheets with RF gels, and d-pyrolysis of the RF-impregnated carbon sheets. CX plates should be made first in the construction of a desalination device. Thus, rectangular-shaped carbon felt sheets (Alfa Aesar GmbH & Co, Karlsruhe, Germany, 99.0%) of (150 × 50 × 6) mm were thoroughly washed with hot deionized water (90 °C) and dried in vacuum at 100 °C for 3 h. The as-prepared carbon felt was impregnated with RF solutions under vacuum at 60 °C for 24 h. Further, the carbon felt containing the RF solution was soaked in ethanol for 3 h and then dried at −40 °C for a 24 h period. The drying process was sustained in an ALPHA 1-2LDplus freeze dryer, CHRIST, Osterode am Harz, Germany. The obtained dried RF-carbon felt sheets were heated between 850 and 900 °C in a controlled atmosphere under argon flow in a tube furnace for 4 h. The flow rate of the inert gas was 20 cm^3^/min. The schematic representation of the overall CX sheets synthesis process is shown below (Figure 1).

### 2.3. Characterisation of the CX Electrodes—Equipment

The morphology of the CX samples was studied by scanning electron microscopy (SEM) using a Carl Zeiss SMT FESEM-FIB (Scanning Microscope Tunneling Field Emission Scanning Electron Microscope-Focused Ion Beam) Auriga, Oberkochen, Germany type scanner in the following conditions: the tension of acceleration was 10 kV and the magnitude was between 20 and 50 k.

The specific surface area and pore size distribution of the CX electrodes were determined using a surface analyzer AUTOSORB—TD1-Quantachrome, Boynton Beach, FL, SUA, based on the N_2_ adsorption/desorption isotherm. The specific surface area was calculated using the Brunauer–Emmett–Teller (BET) method.

Conductivity variations of NaCl solutions and/or artificial sea water used to evaluate the desalination efficiency of the CDI experimental laboratory system were measured by using a digital multiparameter, Consort C860-Fischer Scientific, Merelbeke, Belgium.

A potentiostat/galvanostat VoltaLab 40, Radiometer Analytical, Villeurbanne, France with a dynamic EIS (Electrochemical Impedance Spectroscopy) connected to a computer via the VoltaMaster 4 Software interface was operated for the electrochemical measurements.

## 3. Results

### 3.1. Synthesis of RF Gels

**Figure 2:**
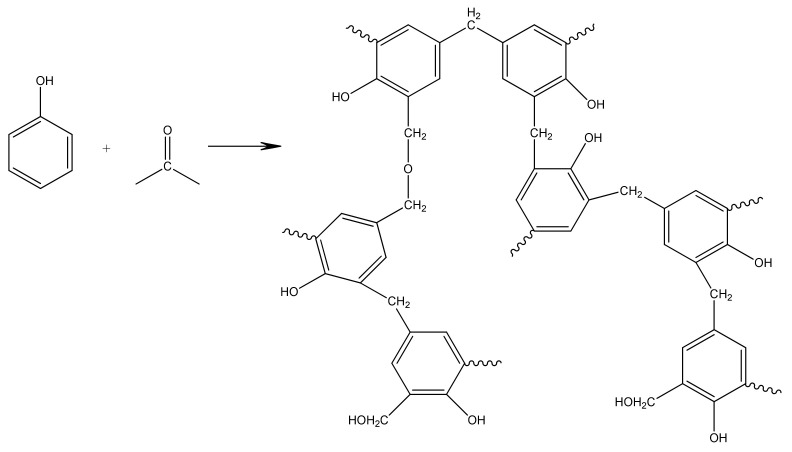
Polycondensation reaction of resorcinol with formaldehyde.

The polycondensation of resorcinol (R) and formaldehyde (F) in the presence of water and sodium carbonate as a catalyst (C) yielded organic RF gels. The aqueous polycondensation of R (1,3 dihydroxybenzene) with F is usually the first step in the synthesis of RF gels. Under alkaline circumstances, this reaction undergoes a sol-gel transition, creating a strongly cross-linked polymer. R acts as a trifunctional monomer in this process, capable of electrophilic aromatic substitution at the active two, four, and six ring locations. Formaldehyde forms a covalent bond between the resorcinol rings, resulting in significant crosslinking densities. The generation of hydroxymethyl (-CH_2_OH) resorcinol derivatives and the condensation of hydroxymethyl derivatives to create methylene and methylene ether-bridging compounds are the main processes as is shown in Figure 2.

**Figure 3:**
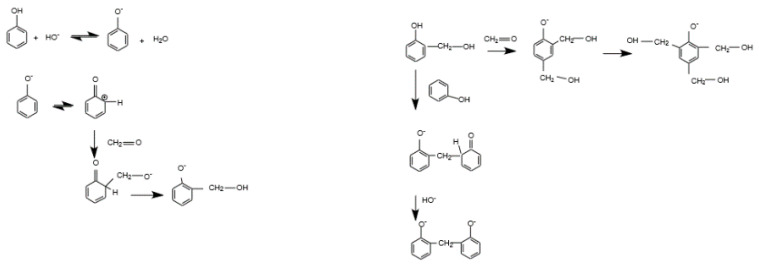
The general mechanism for the development of RF gel networks. Adapted from S.A. Al-Muhtaseb [44].

According to the literature [44] the RF polycondesation mechanism consists of two steps:

1. the synthesis of hydroxymetyl derivates from resorcinol anions via hydrogen abstraction (increased by OH-) and formaldehyde addition;
2. condensation of hydroxymetyl derivates and cluster growth. These two sections demonstrate how the final texture is influenced by the RF gels’ synthesis parameters at the start (e.g., pH and dilution ratio). The first addition reaction is favored when the pH rises, resulting in very branched and unstable aggregates and smaller, more connected polymer particles. In Figure 3 is illustrated the general mechanism of RF gel networks development including formation and condensation of hydroxymethyl derivates.

The formation of nucleation sites, their expansion, and the cross-linking of polymer chains are all affected by the concentrations of the reagents used. The values of these variables will determine the final polymeric structure. Subsequently, the polymeric structure will affect the expected carbon gel features.

### 3.2. Characterizations of CX

In order to assess the effect of the synthesis conditions on the final surface area of the pyrolyzed RF gels, four starting compositions were prepared (Table 1).

Because aerogels contain a significant amount of moisture and organic components, theoretically, they have a low surface area and a high electrical resistance, limiting their use as electrode materials. Pyrolysis removes the volatile materials and facilitates the generation of conjugated sp^2^-carbon atoms, which could be a viable way to enhance the surface area and improve the electrical conductivity. The pyrolysis parameters (e.g., pyrolysis temperature, heating rate, and final temperature) have a significant impact on the textural characteristics (e.g., conductivity, morphology, porosity, and surface chemistry) as is seen in Table 1. As a result, the R/C molar ratio was chosen as the variable that would be held responsible for the polycondensation outcome (the R/F = 0.5 was maintained constant). Therefore, an additional set of variants were prepared to demonstrate the importance of R/C and the dilution ratio on the RF–CX characteristics.

The dilution ratio was considered as: D = water/(resorcinol + formaldehyde + sodium carbonate) molar ratio. Table 2 summarizes the various synthesis parameters.

It became clear that the surface area of RF organogels and CX could be controlled by modifying the synthesis conditions. From the preliminary results shown in Table 2, it became clear that the surface area of an RF gel increases when the R/C ratio is low.

On the other hand, the same R/C and a higher R/F ratio resulted in an increase in the surface area of the organogels and a decrease in the surface area of CX.

**Figure 4:**
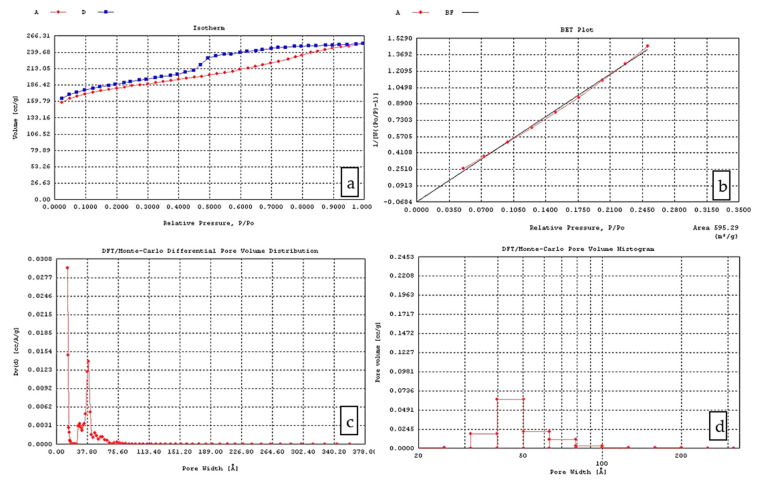
CX Variant X2 (after gel impregnation in the carbon felt and pyrolysis): (**a**) Adsorption isotherm; (**b**) BET plot; (**c**) Pore volume distribution; (**d**) Pore volume histogram.

**Figure 5:**
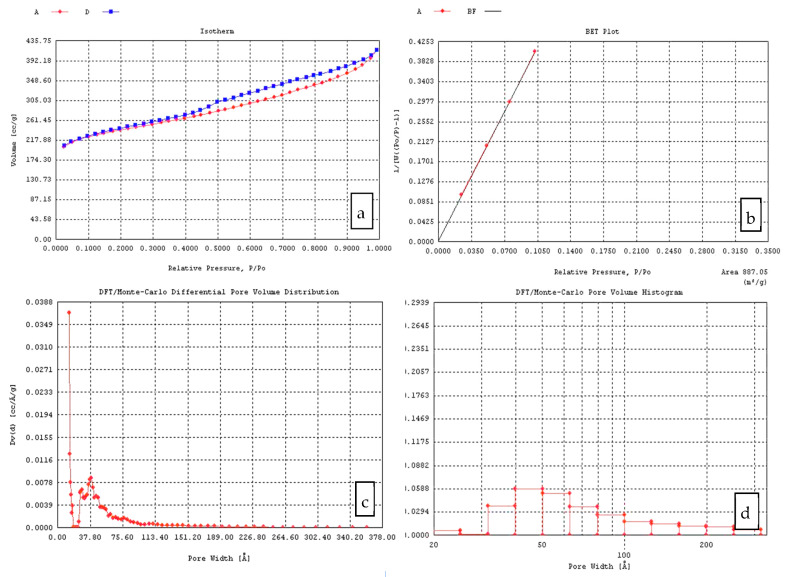
Variant X4 (after gel impregnation in carbon felt and pyrolysis): (**a**) Adsorption isotherm; (**b**) BET plot; (**c**) Pore volume distribution; (**d**) Pore volume histogram.

The pyrolysis temperature also has a strong impact on the surface area of CX. For the same R/C and R/F ratio, the increase in the pyrolysis temperature led to a significant increase in the surface area of the carbon products from 1.03 × 10^2^ m^2^/g to 2.45 × 10^2^ m^2^/g or from 1.55 × 10^2^ m^2^/g to 3.85 m^2^/g. It can be considered that a microporous system is formed during the pyrolysis of RF aerogels. In this view, further investigation regarding the type of evolved porosity must be completed. Figure 4a and Figure 5a show the adsorption–desorption isotherms of the X2 and X4 CX variants. For these samples, type IV isotherms were observed, indicating the presence of mesopores and macro-pores, which correlated with the pore volume measurements (DFT pore volume diagrams) (Figure 4c and Figure 5c) and the pore volume histograms (Figure 4d and Figure 5d).

Type IV isotherms resemble type II isotherms but additionally, instead of adsorption on open surfaces at high relative pressures, adsorption takes place in mesoporosity. Adsorption using mesoporous materials is often associated with a hysteresis loop between the adsorption and desorption isotherm [54].

S_BET_ ranged from 310 m^2^/g (pH = 5.55) to 887 m^2^/g (pH = 6.85). The smallest surface area was registered at the lowest pH and D values, while the biggest surface area value was achieved at the highest D values. The pH and dilution ratio are two obvious variables that could affect the porosity nature of RF gels. However, the pyrolysis process also impacts the surface area by decomposing the organic skeletal system.

### 3.3. Appearance of the RF Gels

The RF solution changed color from colorless to dark red as the polycondensation reaction progressed. The R/C, R/F, or D ratio appears to affect the color of RF aqua gels. For 20 < R/C < 500, the gels were reddish and clear. They became darker as the R/C decreased.

**Figure 6:**
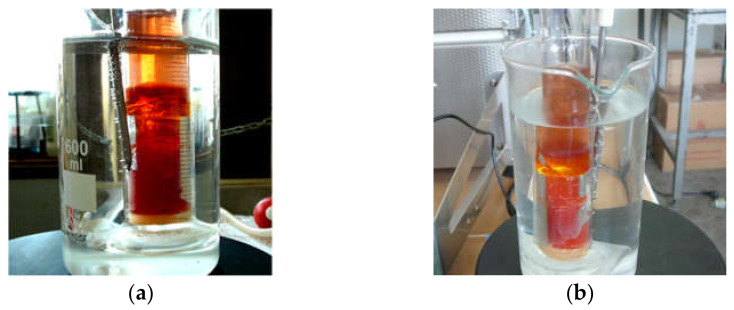
Appearance of RF gels: (**a**) R/C = 500, (**b**) R/C = 200.

In Figure 6 it is shown appearance of an RF gel obtained for (a) R/C = 500 and (b) R/C = 200.

For R/C > 500, the gels were dark reddish in color. The gel’s appearance was also influenced by the R/F ratio. The carbon materials developed as a result of pyrolysis were black. This article did not investigate R/D dependency.

### 3.4. Morphology of the RF Gels after Pyrolysis

**Figure 7:**
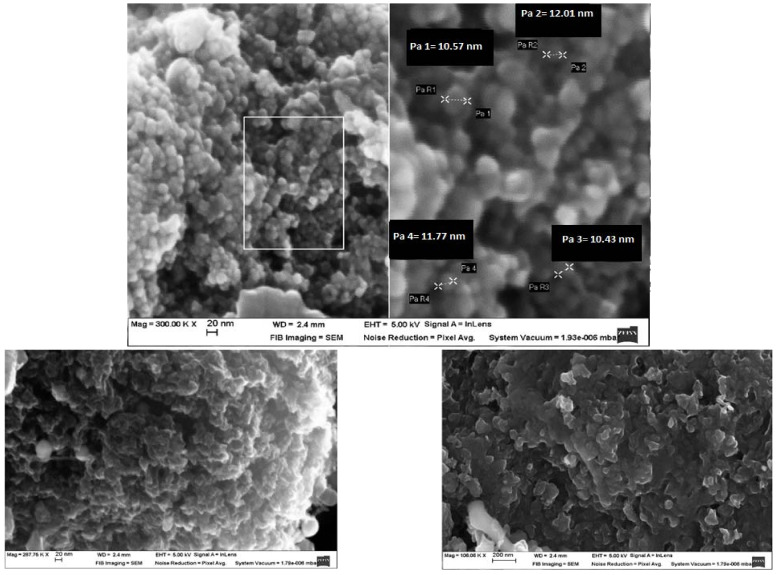
Scanning electron microscopy of the X2 variant (RF gel impregnated in carbon felt followed by pyrolysis).

**Figure 8:**
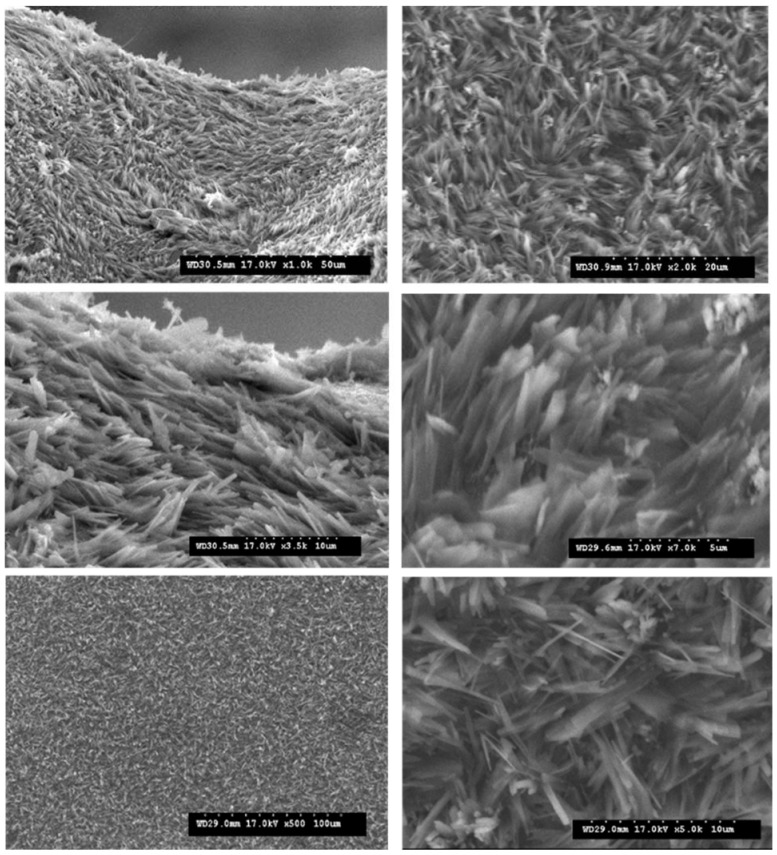
Carbon nanowhiskers. Variant X4 (RF gel impregnated in carbon felt after pyrolysis).

For the morphological analysis, the CX variants with the highest surface area value were considered. Two primary and distinct types of morphologies were obtained based on the synthesis variables. The scanning electron microscopy (SEM) analysis showed that RF–CX could have a structure that is made up of interconnected spherical particles with a hierarchical porosity as is shown in Figure 7 (case of variant X1 and X3) or a randomly aligned whisker morphology as is illustrated in Figure 8. (case of X2 and X4 variants).

It is worth noting that well-developed methods such as laser evaporation, chemical vapor deposition (CVD), arc discharge, and the plasma-controlled environment are generally used to prepare whisker structures [55]. Graphite whiskers are the first nonplanar graphitic structures to be synthesized in a controlled manner [56]. Graphite whiskers and filaments have also been shown to evolve during the pyrolytic deposition of numerous different hydrocarbon materials [57]. In this research, whiskers were generated without the need for a metal catalyst or an external carbon supply. If the surface area values are analyzed, it becomes clear that the X2 and X4 variants that exhibited a whisker type morphology had the highest surface area value. In Figure 7 a scanning electron microscopy of the X4 variant (X4/RF gel variant impregnated in carbon felt) after pyrolysis is shown.

**Figure 9:**
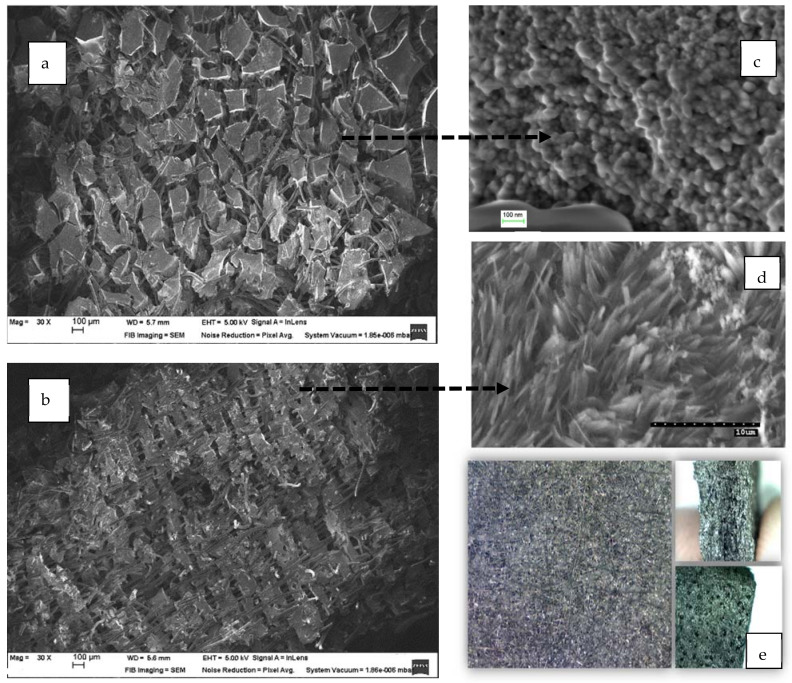
Various morphologies of CX: (**a**,**c**) scanning electron microscopy of the CX electrode X2 variant; (**b**,**d**) scanning electron microscopy of CX type nanowhiskers electrode—X4 variant; (**e**) optical micrograph of the electrode surface × 300.

Pure CX are too brittle to be machined in certain specimen dimensions for an electrode development. The brittleness of the CX was overcome by strengthening it with carbon felt. Combining the flexibility of carbon felt, our carbon felt/CX electrodes could be manufactured in a large-scale environment and machined to any shape, which is critical for this application. As shown in Figure 9e an optical microscopy revealed the appearance of the CX electrode surface.

### 3.5. Electrochemical Characterization of CX-Based Electrodes

**Figure 10:**
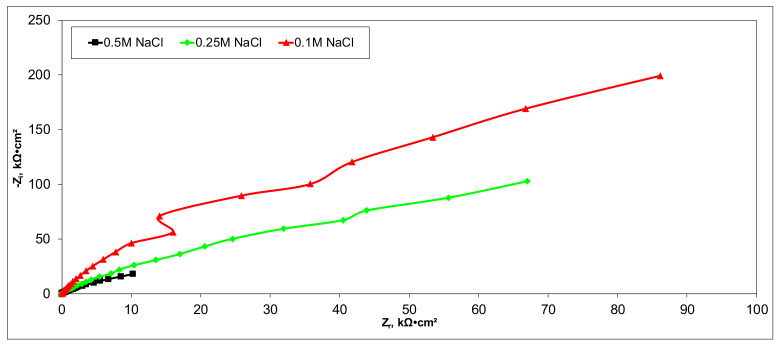
Nyquist plots for the X1 variant in NaCl: 0.5M, 0.25M, 0.1M.

**Figure 11:**
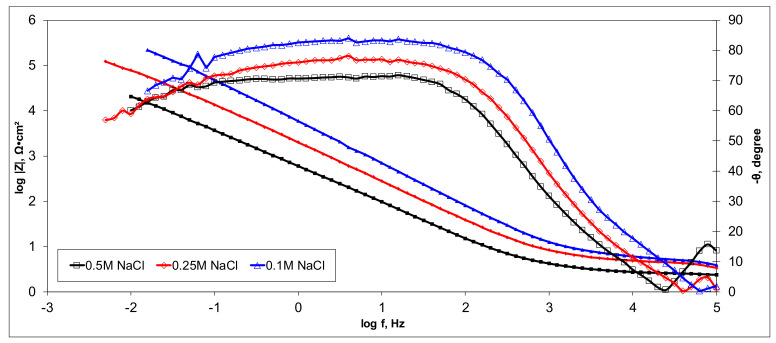
Bode plots for the X1 variant in NaCl: 0.5M, 0.25M, 0.1M.

**Figure 12:**
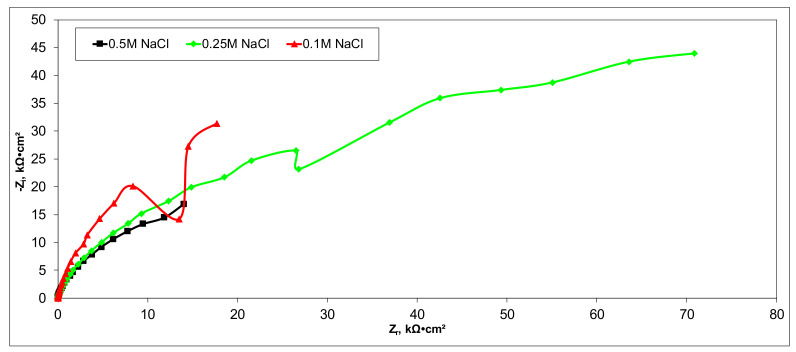
Nyquist plots for the X2 variant in NaCl: 0.5M, 0.25M, 0.1M.

**Figure 13:**
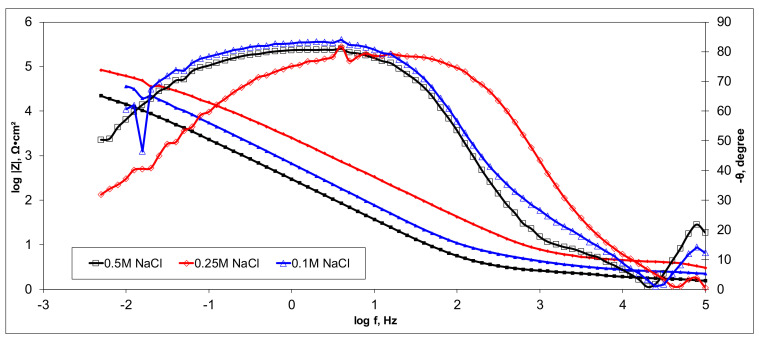
Bode plots for the X2 variant in NaCl: 0.5M, 0.25M, 0.1M.

**Figure 14:**
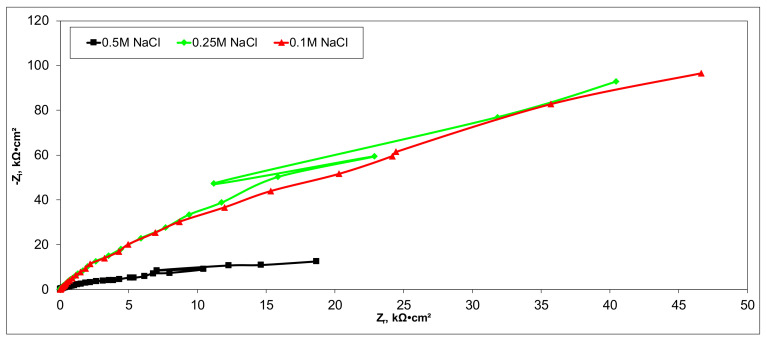
Nyquist plots for the X3 variant in NaCl: 0.5M, 0.25M, 0.1M.

**Figure 15:**
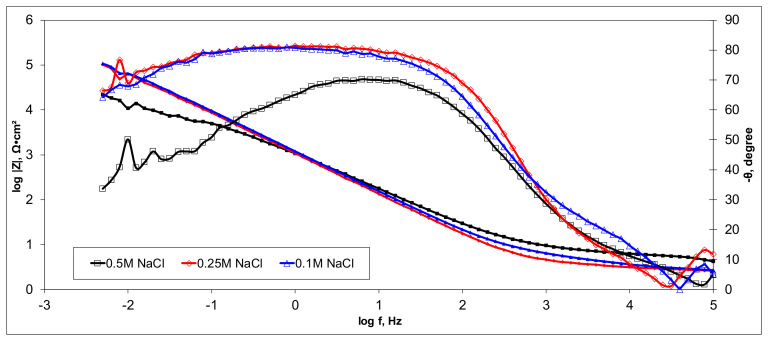
Bode plots for the X3 variant in NaCl: 0.5M, 0.25M, 0.1M.

**Figure 16:**
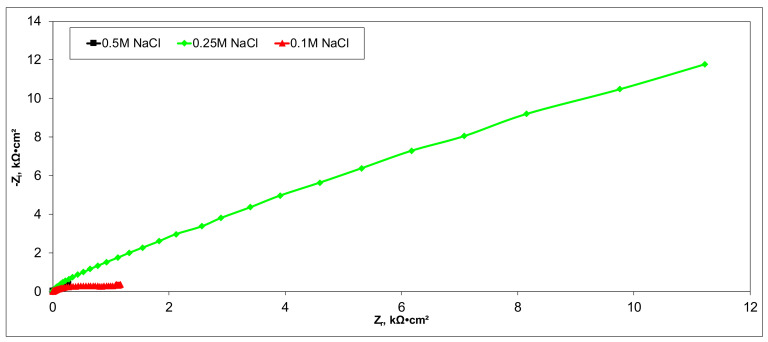
Nyquist plots for the X4 variant in NaCl: 0.5M, 0.25M, 0.1M.

**Figure 17:**
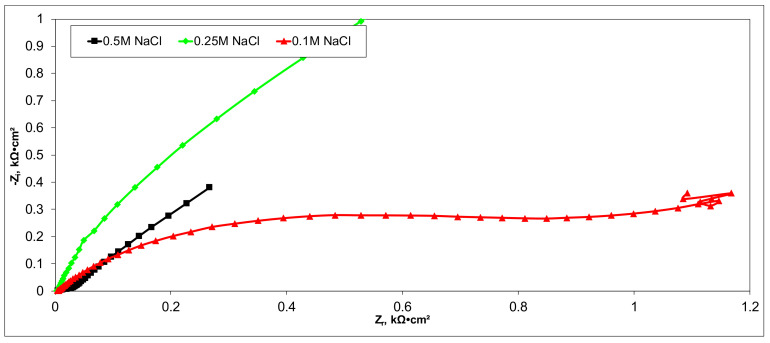
Nyquist (zoom) plots for the X4 variant in NaCl: 0.5M, 0.25M, 0.1M.

**Figure 18:**
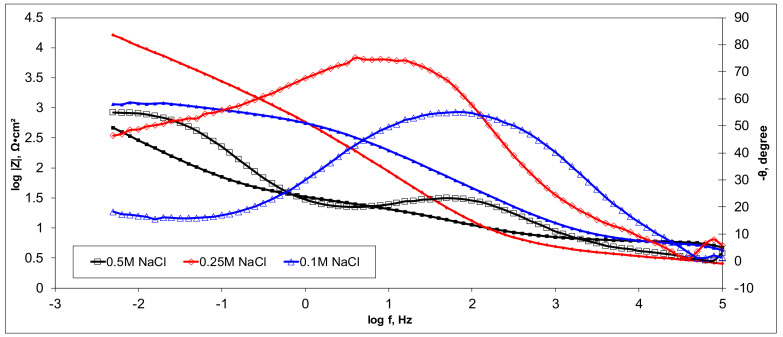
Bode plots for the X4 variant in NaCl: 0.5M, 0.25M, 0.1M.

Electrochemical measurements were performed to select the appropriate CX variant to be further tested as the electrode in our experimental CDI module. The highest recorded double layer capacity was used to consider this selection. Therefore, the electrochemical behavior of the X1, X2, X3, and X4 CX variants was studied in a standard electrochemical cell with a three electrodes arrangement. A platinum plate electrode and an Ag/AgCl electrode were used as a counter and reference electrode, respectively. Samples with a 1 cm^2^ geometric surface area were analyzed. Impedance measurements were performed at the open circuit potential of each sample in the frequency range between 100 kHz and 10 mHz with an AC wave of ±10 mV (peak-to-peak) overlaid on a DC bias potential. The impedance data were obtained at a rate of 10 points per decade change in frequency. All tests were carried out in various NaCl solutions with concentrations of 0.5M, 0.25M, and 0.1M at room temperature and 1012 ± 5 hPa under atmospheric conditions, without agitation. Bode and Nyquist plots for the X1–X4 carbon xerogel variants are illustrated below (Figure 10, Figure 11, Figure 12, Figure 13, Figure 14, Figure 15, Figure 16, Figure 17 and Figure 18).

A circular regression with a fitting coefficient greater than 0.98 was used to derive the EIS parameters such as, electrolyte resistance (R_el_), polarization resistance (R_p_), and double layer capacity (C_dl_) from the Nyquist plots (Table 3).

Except for sample X2 in NaCl 0.25M, the polarization resistance fell as the electrolyte concentration increased in all situations. Furthermore, as the electrolyte concentration dropped, the capacity of the double layer decreased.

Of all the electrolyte concentrations, the X4 sample (carbon nanowhisker structures) had the highest double layer capacity and the lowest polarization resistance. At all electrolyte concentrations, the Bode plots show a general medium-high capacitive behavior with medium-weak diffusive tendencies (phase angle values were between 65 and 800) associated with a single time constant in the case of the X1, X2, and X3 samples and a single Debye semicircle on the Nyquist plots. A distinct response was found for the X4 sample, which is dependent on the electrolyte concentration. At 0.5M we can see a two-time constant evolution (a duplex equivalent of two distinct electrode–electrolyte interfaces) in the frequency range, first at 100 Hz associated with an inductive behavior (corresponding phase angle at approx. 250), followed by a second time constant at 10mHz (corresponding phase angle at approx. 550) associated with a weak capacitive behavior with high diffusive tendencies. In the case of the 0.25M NaCl electrolyte, the X4 sample showed a single time constant at a frequency around 10 Hz with a phase angle near 800, while in the case of 0.1M NaCl electrolyte, the single time constant was situated at 100 Hz and a phase angle of 550. This type of non-linear variable behavior in the same electrolyte at different concentrations suggested that the electrode–electrolyte interface(s) were subjected to different phenomena in the case of X4 sample. The synthesis parameters applied to obtain the X4–CX type nanowhiskers variant are reflected in a surface area that was higher than the other samples (887 m^2^/g) (Table 2). As a result, the electrode–electrolyte interface may have a higher hydrophilic behavior, resulting in a significant increase in the electrochemical surface area and double layer capacity (Table 3).

### 3.6. Performance Evaluation of CX-Based Electrodes for Capacitive Desalination

#### 3.6.1. CDI Operating Principle

A CDI cell is made up of two porous electrodes, isolated by a non-conductive material. The feed stream flows between or through the charged electrodes [58]. Desalination is accomplished by exposing the electrodes to a low voltage, between 0.8 and 1.5 V, which causes the salt ions in the feed stream to move to the electrical double layers (EDLs) at the carbon—water interface [59]. When the electrodes become saturated, the electrical potential can be reversed (the anode becomes the cathode and conversely) releasing the adsorbed ions from the electrodes.

#### 3.6.2. Design of Laboratory Scale CDI Module

The CDI module, used in our study to assess the desalination potential of the CX-based electrode, consists of 11 cells formed by pairs of electrodes. Each cell includes two CX type nanowhisker electrodes of (150 × 50 × 6) mm^3^ each, arranged in a parallel plane, equidistant with a spacer (non-conducting membrane) in between. The separator media is necessary to ensure electrical isolation between electrodes and the facile flow of the water stream through the CX electrodes.

**Figure 19:**
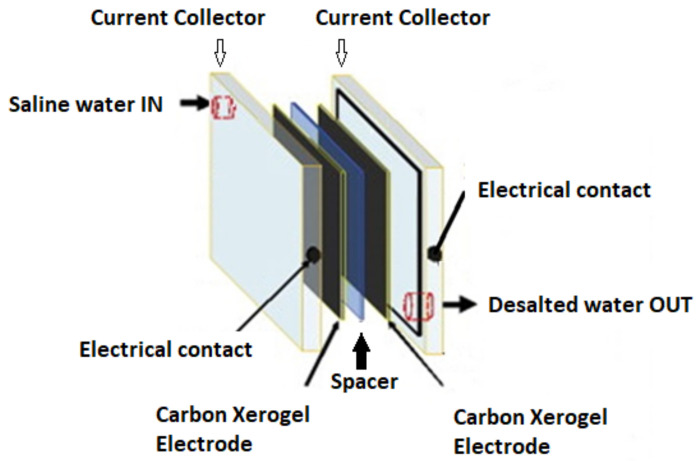
Schematic configuration of one CDI cell.

The carbon electrodes are charged by means of collectors, with the help of a stabilized DC voltage source. Rectangular plates (of the same size/geometrical features as CX electrodes) made of synthetic graphite (electrographite-EG122-Electrographite Carbon CO.LTD) were used as current collectors. Through electrical connections, the collectors are in direct contact with the power source. The basic CDI unit cell has an electrode separation distance of 1.5 mm. Figure 19 shows a schematic diagram of one CDI cell.

The CDI laboratory module was constructed by stacking 11 CDI unit cells. Overall, the CDI module comprised: 22 CX type nanowhisker-based electrodes, 20 electrographite electrical current collectors, and 20 spacers.

**Figure 20:**
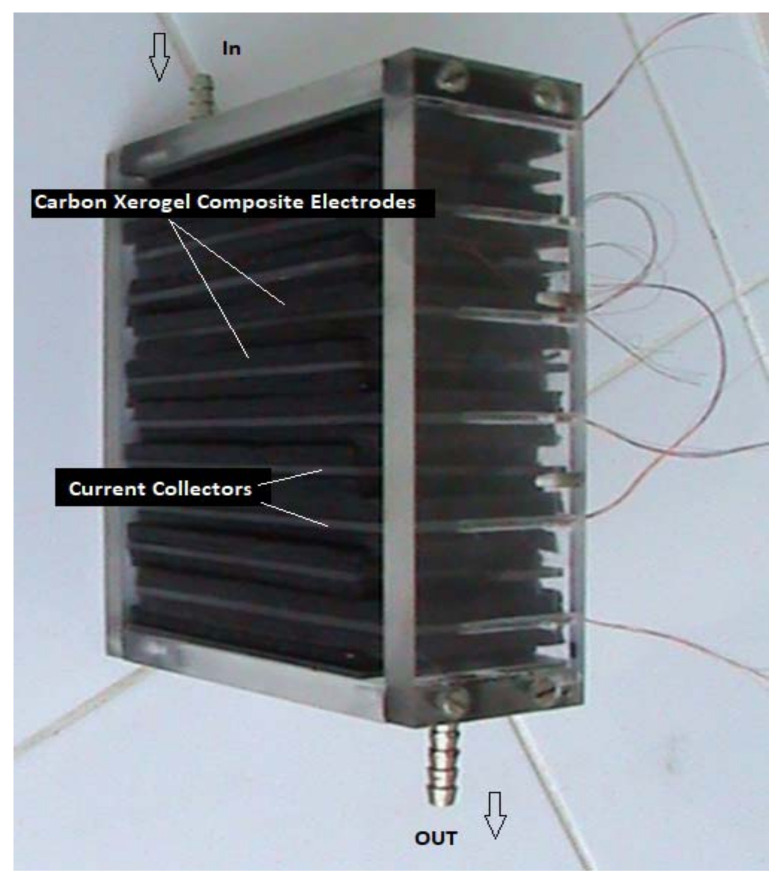
Cross image of the CDI stack module-based CX type nanowhisker electrodes.

An image of the constructed CDI stack-based CX-type nanowhiskers is illustrated in Figure 20.

The water feed was injected into the CDI module using a peristaltic pump (DC 24V Motor No. KDM 3429-24-500, Kookjae Precision Co., Incheon, Korea). As the saline feed stream in this study, NaCl 0.5M solution and artificial sea water were used. When the electrodes are subjected to a low voltage (0.8–1.2 V) during the operating phase, cations and anions are drawn towards them and adsorbed until they become saturated. Due to the electrodes’ limited ion adsorption capacity, the number of ions transported and adsorbed on the electrode surface gradually decreases over the operation cycle. As a result, the flowing water stream conductivity steadily increases up to the inlet water’s conductivity. The experiments were conducted continuously (without water re-circulation). Controlling the potential difference across each cell at a constant voltage is required. As a result, the electrode pair wiring layout inside the module was chosen to have the electrodes connected in parallel. The use of parallel electrodes has a number of advantages such as: a. the system can be easily adjusted at the necessary low voltage values; b. it is safer from an operational perspective due to the voltage setup; c. all electrode pairs have uniform operational conditions, resulting in a more homogeneous ion adsorption setting. The composition of the artificial sea water used in the experiments is shown in Table 4.

**Figure 21:**
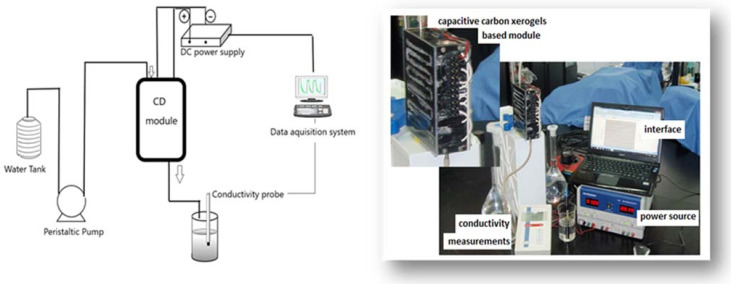
Schematic diagram of the experimental set-up of the laboratory CDI system.

A schematic diagram of the CDI experimental set-up next to an image of the laboratory CDI system is shown below (Figure 21).

As previously stated, the tests were carried out with two types of aqueous streams: NaCl solutions and artificial seawater at voltages ranging from 0.6 V to 1.2 V.

The output of the desalination was calculated using formula (1).

(1) $$\eta (\%)=\frac{C_{i}-C_{f}}{C_{i}}\times 100$$

where:

η—desalination yield.
$C_{i}$—initial conductivity of the feed stream.
$C_{f}$—conductivity at the output of the CDI module.

The charging cycle of the desalination process was the only one tested. Thus, at an optimum water flow of 1.5 L/h, the experimental CDI module could perform desalination for up to 2 h.

**Figure 22:**
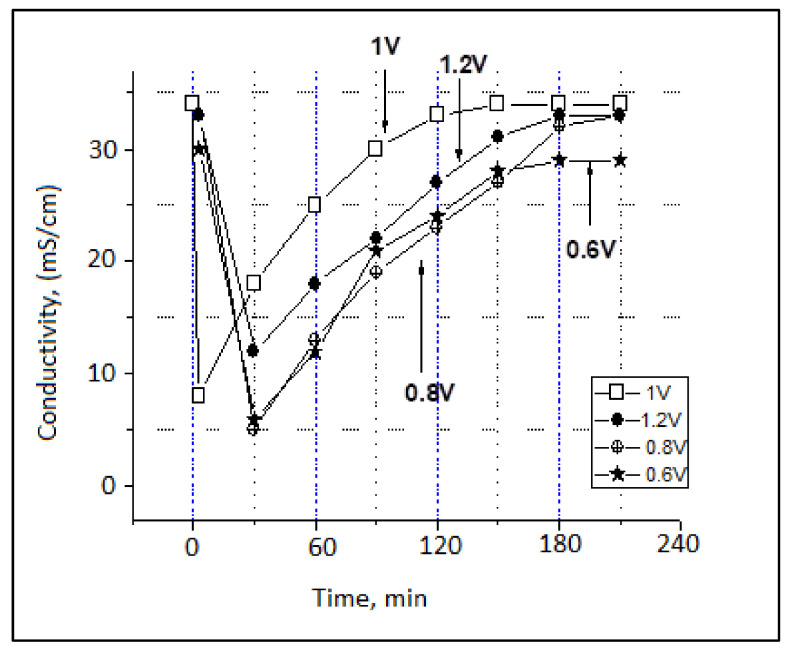
Variation of ionic conductivity of the NaCl test solution during the charge cycle.

**Figure 23:**
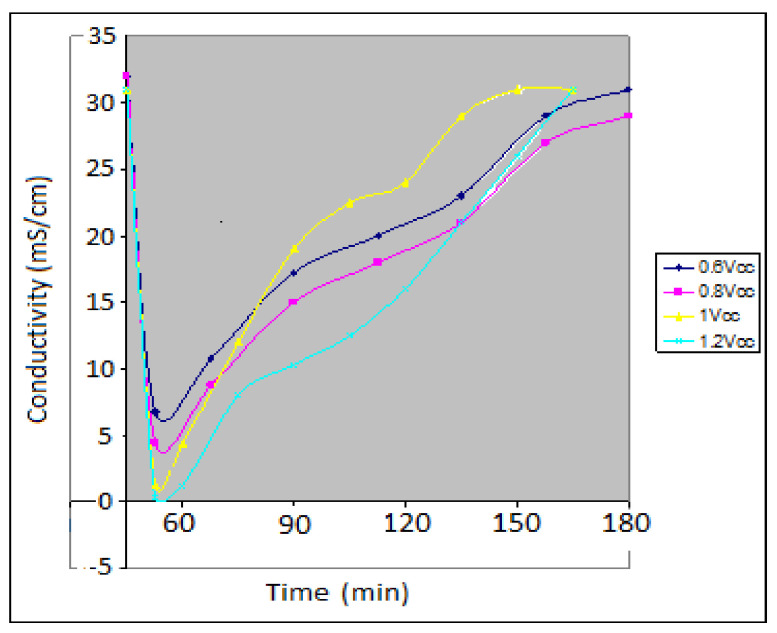
Variation of the ionic conductivity of the artificial sea water test solution during the charge cycle.

The ionic conductivity of the test solution was approx. 35 mS. As is shown in Figure 22 and Figure 23, the desalination process occurs almost instantly when the water passes through the CDI module.

Ionic conductivity dropped by up to 5–8 mS in the first hour of the operation in the case of the NaCl test solution and by up to 65 µS in the case of the artificial seawater. Furthermore, the deionization/desalination process took longer in the case of artificial seawater (180 min until saturation) than in the case of the NaCl solution (120 min until saturation).

Therefore, the CDI module is thought to operate optimally for multicomponent aqueous mixtures (seawater case). After the first hour of operation, the ionic conductivity of the outlet effluent began to gradually increase until saturation was reached.

The process took about 2 h for a 3 L water volume to attain saturation (roughly 1.5 L per hour). The 1 V supply voltage is considered to be optimal.

Under these conditions, the estimated energy consumption is:

(2) $$W=P\times t=U\times I\times t\;\left[\mathrm{Wh}\right]$$

W— energy consumption, Wh
U— voltage, V
I— recorded electrical current, mA: I = 290 mA
t— time, h

W=1 V×0.290 A×2 h=2.29 Wh

and, current density:

(3) $$J=\frac{\mathrm{dI}}{\mathrm{dS}}\;[A/m^{2}]$$

where, the total geometrical surface of the electrodes, S = 0.132 m^2^

$$J=\frac{\mathrm{dI}}{\mathrm{dS}}=\frac{290\;\mathrm{mA}}{0.132\;m^{2}}=2.196\;A/m^{2}$$

Table 5 shows that the desalination yields varied between 63.63% and 99.09% percent depending on the supply voltage and the type of solution tested (mono or multicomponent).

## 4. Conclusions

This study aimed to demonstrate that materials such as carbon nanowhiskers-derived organic gels have real potential to be used in CDI applications. Carbon whiskers have never been used as electrode materials in CDI, as far as we know. These structures were generated using the same chemical processing path as carbon xerogels, rather than a plasma-controlled environment or any other commonly used physical method. In this regard, the processing method of the carbon whiskers will tend to reflect the cost of electrode manufacture, becoming cost effective. Laboratory trials confirmed that carbon nanowhiskers can compete from an operational perspective with well-known porous carbon xerogels. Given the high desalination yield of 99.09% obtained when using artificial seawater solutions, the desalination rate experimental trials have shown that the CDI module-based carbon whiskers are expected to work best with multi-component aqueous solutions or concentrated streams. The research is still ongoing to improve the electrode’s efficiency and surface area by refining the factors responsible for the electrode’s functional properties and to reach more reliable results for water desalination.

## 5. Patents

RO129080B1 Process for Preparing A Composite Carbonic Material for Capacitive Desalinization of Sea Water, Hristea Gabriela, Lipcinski Daniel, Militaru Adrian Gigi.

## Figures and Tables

**Table 1:** Synthesis parameters for RF carbon xerogels.

Reagents Molar Ratio	Temperature of Pyrolysis Process, (°C)	Organic Gel/ Carbon Xerogel	S_BET_ (m^2^·g^−1^)
R/C = 20 R/F = 0.5	800	RF gel CX	7.25 × 10 1.03 × 10^2^
R/C = 200 R/F = 0.5	800	RF gel CX	4.23 × 10 1.55 × 10^2^
R/C = 20 R/F = 0.5	950	RF gel CX	7.25 × 10 2.45 × 10^2^
R/C = 200 R/F = 0.5	950	RF gel CX	2.85 × 10 3.85 × 10^2^

**Table 2:** The synthesis parameters of carbon xerogels.

Variant	pH	R/C	D (Dilution Ratio)	Na_2_CO_3_ (g)	S_BET_ (m^2^·g^−1^) RF Gels after Pyrolysis	S_BET_ (m^2^·g^−1)^) Carbon Felt Impregnated with RF Gels after Pyrolysis
X1	5.5	1000	0.91	0.035	290	310
X2	6.04	500	0.95	0.077	267	595
X3	7.55	20	0.96	0.773	209	485
X4	6.85	1000	1.80	0.031	315	887

**Table 3:** EIS parameters for X1, X2, X3, and X4 CX-based electrode variants.

Variant	Electrolyte	R_el_, kΩ·cm^2^	R_p_, kΩ·cm^2^	C_dl_, μF/cm^2^
X1	NaCl 0.5M	1.54 × 10^−3^	100.5	158.3
	NaCl 0.25M	86.9 × 10^−3^	460.4	69.13
	NaCl 0.1M	39.9 × 10^−3^	851.5	11.81
X2	NaCl 0.5M	8.93 × 10^−3^	41.65	603.6
	NaCl 0.25M	215 × 10^−3^	140.6	226.3
	NaCl 0.1M	8.76 × 10^−3^	111.4	71.39
X3	NaCl 0.5M	13.4 × 10^−3^	44.44	716.1
	NaCl 0.25M	42.19 × 10^−3^	377.2	84.38
	NaCl 0.1M	56.9 × 10^−3^	461.3	69
X4	NaCl 0.5M	5.57 × 10^−3^	32 × 10^−3^	777.9
	NaCl 0.25M	28.1 × 10^−3^	72.68	437.9
	NaCl 0.1M	3.71 × 10^−3^	1	158.8

**Table 4:** Artificial sea water composition.

Component	Molecular Weight	g/L	
NaCl	58.44	23.936	
Na_2_SO_4_	142.04	4.008	
KCl	74.56	0.677	
NaHCO_3_	84	0.196	
KBr	119.01	0.098	
H_3_BO_3_	61.83	0.026	
NaF	41.99	0.003	
	Molecular weight	Mol/L solution	Concentration
MgCl_2_ × 6H_2_O	203.33	0.05327	1.0 M
CaCl_2_ × 2H_2_O	147.03	0.01033	1.0 M
SrCl × 6H_2_O	266.64	0.00009	0.1 M

**Table 5:** CDI module desalination yields for the test solution at different applied voltages.

NaCl Solution		Artificial Sea Water	
**Voltage,** **V**	**Desalination Yield,** **%**	**Voltage,** **V**	**Desalination Yield,** **%**
1.2	63.63	1.2	99.09
1.	76.47	1.	96.12
0.8	84.85	0.8	86.65
0.6	79.31	0.6	85.62

## Data Availability

Not applicable.
